# The role of ponds in the retention of micro(nano)plastics, tyre wear particles and their leachates: current state of the science, research gaps and opportunities

**DOI:** 10.1039/d6ew00639f

**Published:** 2026-10-19

**Authors:** Stuart Cairns, Muhammad Junaid, Iain Robertson

**Affiliations:** a Department of Geography, Swansea University Swansea Wales UK S.L.Cairns@Swansea.ac.uk

## Abstract

Natural and artificial ponds offer a cost-effective management technique to potentially remove water contaminants including micro(nano)plastics (MNPs) and tyre wear particles (TWPs). The use of sustainable urban drainage systems (SuDS), including ponds, is increasingly being promoted by governmental guidance in countries such as the UK, the USA, and Denmark in part to address contaminated runoff in new road and housing developments with increasing calls to make these systems mandatory. Traditionally, the focus has been on the removal of contaminants such as metals, suspended particles, PAHs and pesticides, improving water quality and reducing diffuse pollution. In more recent years, the efficacy of ponds in the removal of MPs and TWPs has been increasingly investigated. Studies estimate, albeit with potentially substantial uncertainty, that pond removal efficiencies for microplastics (MPs) and TWPs range from 83.9% to 99.9% and 80.1% to 100.0%, respectively. However, with current legislative steps toward ponds becoming increasingly widespread as a remediation tool, there are a number of significant gaps in the current body of research that need to be addressed. The transport and fate of nanoplastics (NPs) need to be examined, TWPs need to be consistently included in studies, and the transport through ponds of contaminants sorbed by MNPs and TWPs alongside manufacturing additives leached by MNPs and TWPs must be considered. As analytical techniques develop and are standardised, future studies should ensure that these aspects are considered more thoroughly to enable relevant conclusions to be drawn that could shape the design of future pond systems.

## Abstract 2

Ponds offer a cost-effective management technique to potentially remove water contaminants including micro(nano)plastics (MNPs) and tyre wear particles (TWPs), however challenges around the transport of additives and sorbed contaminants remain.

## Graphical abstract

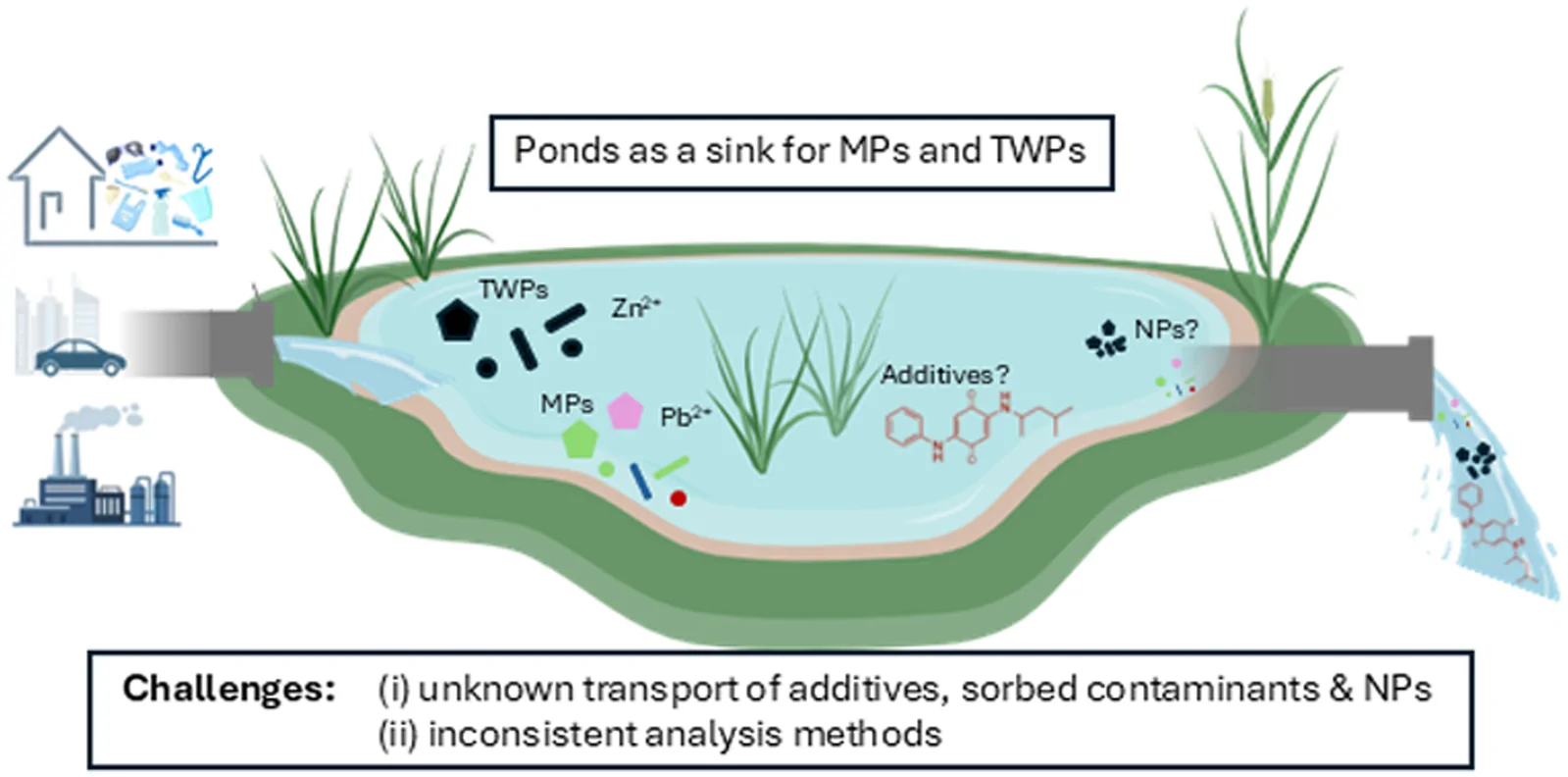

**Water impact**

Ponds offer a cost-effective technique to remove water contaminants including microplastics and tyre wear particles. They are increasingly advocated by governmental guidance in new developments, increasing adoption. This review outlines the current understanding of pond efficacy regarding microplastics and tyre-wear-particle removal. Gaps in the literature are highlighted that must be considered in future pond design including analytical limitations and plastic-additive and nanoplastic retention.

## 1. Introduction

The production and use of plastics have been increasing exponentially since the advent of mass production in the 1950s with plastic products becoming indispensable to the contemporary world.^1^ The global annual production of plastics rose to ∼430 Mt in 2024 with production projected to double by 2050.^2,3^ Plastic litter, including the resultant micro(nano)plastic (MNP) degradation products, is a constantly increasing global issue representing a key environmental challenge.^4^ These MNPs are varied in size, shape and chemical composition and whilst there is not a consistent nomenclature for the sizes of these degraded plastics, it is generally recognised that the size fraction of microplastics (MPs) is between 5 mm and 1 μm and the size fraction of nanoplastics (NPs) is below 1 μm.^5^ Of these plastics, the most widely used and fabricated polymers include polypropylene (PP), polyethylene (PE) including both low-density polyethylene (LDPE) and high-density polyethylene (HDPE), polyvinyl chloride (PVC), polyethylene terephthalate (PET), and polystyrene (PS).^6^ PE, PP, PET and PS are often used for single-use packaging. PP and PET are widely used in textiles such as bedding and clothing, and PVC is commonly used in building and construction as well as for health care products.^6,7^ It is estimated that ∼60% of this plastic is mismanaged globally and disposed of environmentally with ∼23 Mt a^−1^ being transported to waterways.^8^

In tandem with the growth in plastic production, the number of motor vehicles and the related increase in highway development have resulted in the increased release of tyre wear particles (TWPs) with the potential for associated ecological consequences.^9^ TWPs partly meet the criteria for the International Organization for Standardization's description of plastics as they contain a high polymer as an essential ingredient. However, TWPs are not officially classified as plastics due to their reversible elastic deformation, resulting in them often being overlooked in many studies concerning the quantification of environmental plastics.^10^ Whilst it must be recognised that there are key differences between TWPs and MNPs, the ubiquitous nature of TWPs necessitates their inclusion in environmental studies to provide a holistic perspective. Key distinctions between the materials include differing surface properties, TWPs' more complex chemical composition, and TWPs' density being higher compared to most MPs.^11^ These differences cause MNPs and TWPs to behave differently from each other in water and crucially for each of them to require different analytical techniques to quantify their presence. Nevertheless, when TWPs are considered alongside MNPs as organic particulate contaminants, studies estimate that TWPs account for between 50% and 70% of particulate release into the environment, underlining the importance of their inclusion in environmental studies particularly in relation to environmental degradation and adverse human health effects.^10,12^ The primary source for the formation of TWPs is abrasion between the tyre and the road surface with >99.9% remaining as particulate matter on the road surface with a dispersion primarily ascribed to runoff into the environment.^13^ The size fraction of TWPs can be categorised into coarse (PM10), fine (PM2.5) and ultrafine (PM0.1). TWPs found in road runoff often fall into the coarse and fine categories, *i.e.* from <1 μm to 100 μm.^14^ A quantitative estimate of TWPs generated during road use is ∼2.5 Mt a^−1^ for the EU and the USA combined.^14^

Urban runoff is the primary pathway for the transfer of both MNP and TWP pollution to the aquatic environment.^15^ The transformation of native and agricultural lands to impermeable urban surfaces has profoundly transformed the hydrologic regime with urban surfaces acting as a collection point for pollutants, such as MNPs and TWPs, which are transported to waterways during periods of precipitation in the form of urban runoff.^16,17^ This runoff is often discharged without treatment. However, prior to discharge, ponds can offer a cost-effective management technique to potentially remove water contaminants including MNPs and TWPs. The use of sustainable urban drainage systems (SuDS), including ponds, is increasingly being promoted by governmental guidance in countries such as the UK, Denmark, and the USA in part to address contaminated runoff in new road and housing developments, increasingly stringent water quality targets, and to remove pollutants prior to discharge to rivers.^18–20^ Traditionally, the focus has been on the removal of contaminants such as metals, suspended particles, PAHs and pesticides, improving water quality and reducing diffuse pollution.^21^ However, in more recent years, the efficacy of ponds in the removal of MPs and TWPs has been increasingly investigated. For particulate materials, sedimentation and deposition are the primary retention mechanisms with sorption and (photo)degradation representing the key processes for the removal of soluble contaminants such as biocides or pharmaceuticals.^21,22^ With the focus on MPs and TWPs in ponds being more recent, pond retention mechanisms are less well understood particularly when TWPs and MNPs are considered as vectors for pollutants rather than simply as particulate matter. Environmental weathering processes, such as photodegradation, thermo-oxidative degradation, abrasion and biodegradation, alter the surfaces of MNPs and TWPs. These processes affect MNP and TWP size, behaviour, sorption of and by other compounds and increase the likelihood of the leaching of plastic additives.^23^ The presence of NPs causing deleterious health effects is of particular concern. NPs have been seen to cross human tissue membranes into the blood, promoting uptake with resultant cytotoxicity and genotoxicity and correlation to cardiovascular events and reduced brain function.^2,24–26^ When the environmental degradation of larger plastics into MNPs uncovers previously unexposed surfaces, the effect is twofold, increasing the propensity for the sorption of environmentally found contaminants and enabling the leaching of toxic additives introduced during material production into ecosystems. MNPs in aqueous matrices have been observed to combine with existing aqueous contaminants, such as metals, per- and polyfluoroalkyl substances (PFAS), polycyclic aromatic hydrocarbons (PAHs) or pharmaceuticals, leading to increased accumulation and particle retention in terrestrial and aquatic organisms with the consequence of enhancing toxicity.^27–29^ Whilst there are inconsistencies in tyre composition as a result of manufacturer, age, and seasonality of tyres,^10,30^ there are some common concerns and growing evidence of the deleterious effects of leached plastic additives on health and ecosystems.^31,32^ Studies have demonstrated that TWPs leach contaminants, such as diphenylguanidine (DPG); *N*-(1,3-dimethylbutyl)-*N*′-phenyl-*p*-phenylenediamine (6PPD); its transformation product, *N*-(1,3-dimethylbutyl)-*N*′-phenyl-*p*-phenylenediamine quinone (6PPD-Q); and Zn and Pb into waterways.^33^ With the increase in the use of ponds to mitigate the transport of plastics into waterways, the study of MNPs and TWPs within this natural environment is critical to understanding their fate, transport, environmental impact and human health consequences.

The main objective of this review is to assess the current literature on the efficacy of ponds in retaining MNPs, TWPs, associated leachate products and (de)sorbed contaminants from contaminated runoff. While previous reviews have considered the broader treatment of urban stormwater, such as the review by Stang *et al.* (2022),^34^ there is a need for an updated synthesis that incorporates the growing body of research on TWPs, alongside advances in analytical techniques for their detection and characterisation, enabling more precise and accurate measurements, and the treatment of plastic leachates and (de)sorbed contaminants. Notably, ∼65% of the research published on this topic has emerged between 2022 and 2026, highlighting the rapid development of this field and the need for a comprehensive and up-to-date assessment of the current evidence base. More recent reviews have been presented relating to MPs and stormwater runoff such as those of Ahmad *et al.*^35^ and Kwarciak-Kozłowska and Madeła,^36^ but these reviews have been broad in their approach, discussing a selection of green infrastructure solutions primarily bioretention systems, constructed wetlands, and permeable pavements rather than focusing on ponds. Furthermore, the potential impacts of plastic and tyre-derived additives released through leaching as well as contaminants (de)sorbed from MNPs and TWPs within ponds remain insufficiently considered in both reviews and published research.

This review addresses these gaps by examining the fate and transport of TWPs and NMPs within and through pond systems. We discuss the evidence regarding the extent to which ponds can retain these particles, associated leachates and (de)sorbed contaminants. Finally, we identify critical knowledge gaps and pose key research questions to inform future studies and improve our understanding of how ponds can be used to remediate TWPs and MNPs alongside associated contamination from runoff.

## 2. Methods

A systematic literature review was conducted using multiple databases including Scopus, Science Direct, and Google Scholar. Search strings with different combinations were used which included “stormwater OR retention OR balancing OR infiltration OR basin OR wetland AND pond AND tyre OR plastic (searching Article title, Abstract, Keywords)”. The date of the first literature search was May 2025 and the date of the final literature search was January 2026. No date exclusion was applied to the search criteria. The returned studies then were subjected to a vetting process with selection criteria comprising journal papers that were original research articles and published in English. Initially a total of 29 articles highlighting the role of natural and artificial ponds in the retention of MNPs and TWPs were selected for review. For these 29 a further screening process was adopted to ensure that the research articles included in the review were undertaken at field sites and field sites were either natural or artificial ponds. Studies that related to runoff not captured by ponds were excluded as were studies that focused on SuDS but excluded ponds. The 17 remaining studies were geographically located in Denmark (*n* = 4), USA (*n* = 3), UK (*n* = 2), Canada (*n* = 2), Sweden (*n* = 2), France (*n* = 1), Australia (*n* = 1), Brazil (*n* = 1), and India (*n* = 1).

## 3. Results and discussion

### 3.1 Ponds

Ponds are seasonal or permanent water bodies that have a surface area between 1 m^2^ and 2 ha (20 000 m^2^), which can be either artificial or natural water bodies (Fig. 1).^37^ Man-made ponds, specifically sustainable drainage systems (SuDS), have three key benefits which mimic the benefits of natural ponds: (i) treatment of stormwater pollutants, (ii) attenuation of runoff, and (iii) promotion of biodiversity.^38–40^ Due to the positive attributes of ponds, they are part of legislation that makes them mandatory in new developments in countries such as Scotland and many parts of England and Wales.^18,41^ Traditionally, the study of pond water quality benefits has concentrated on metals, suspended particles, PAHs and pesticides.^21^ However, with the rise in awareness of the deleterious environmental and health effects associated with MNPs and TWPs, pond studies focusing on these contaminants are increasing in number with only 35% of papers on this topic being published prior to 2022 and 65% being published from 2022 to 2026.

**Fig. 1:**
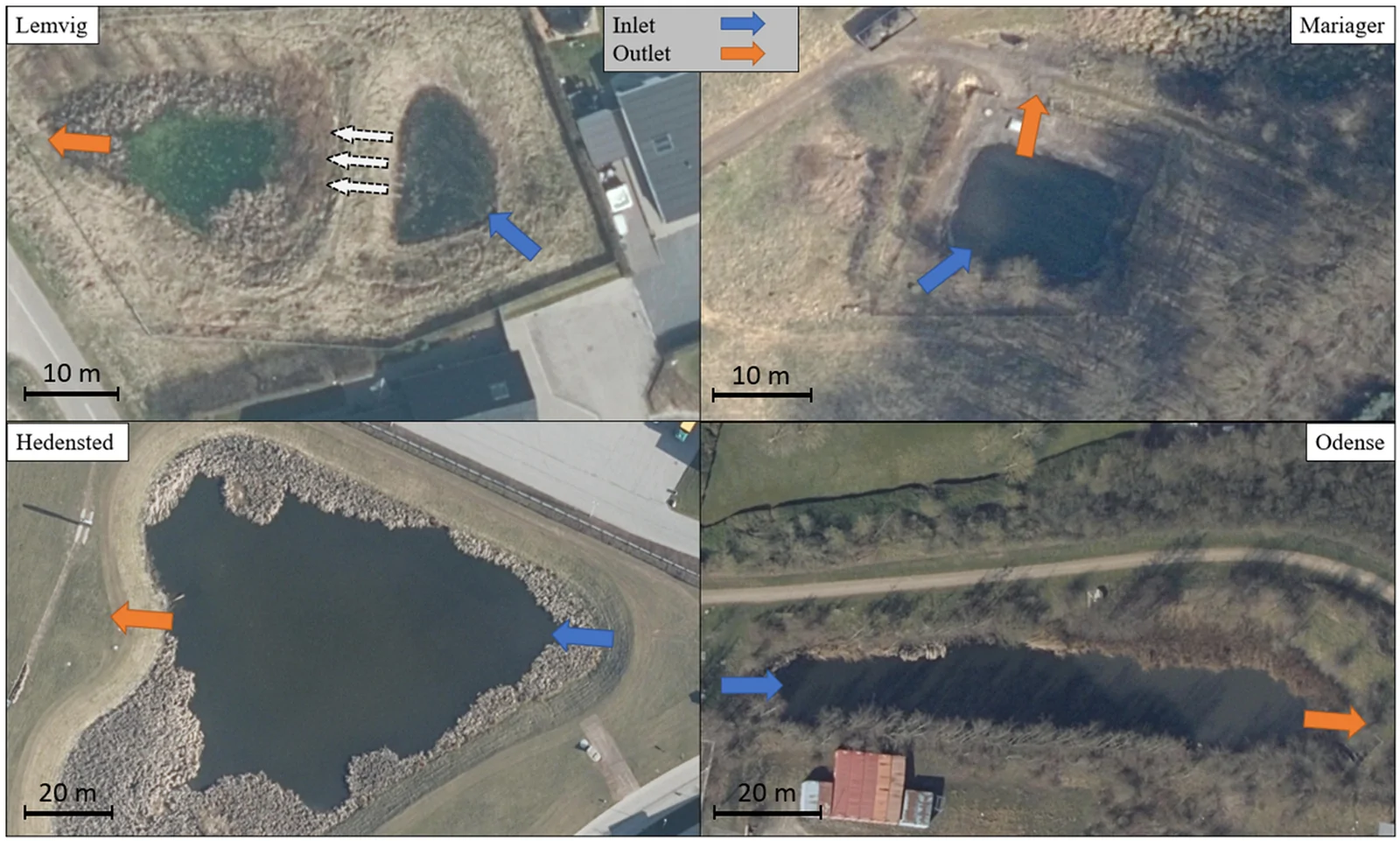
Stormwater retention ponds in Denmark.^45^

### 3.2 Micro(nano)plastic and tyre wear particle analysis

MNP and TWP analysis is a developing field with analysis complicated by the vast array of contaminant shapes, sizes, polymers and colours. Plastics and TWPs are emerging contaminants and as such are a relatively new analytical problem with the word microplastic not even coined until 2004.^42^

#### 3.2.1 Sample matrix

Alongside the variety of polymer and elastomer characteristics, the matrix from which these MNP and TWP pond contaminants are analysed can also vary. Pond matrix selection is generally driven by site characterisation and the objectives of the study, most commonly including sediment and water, with invertebrate contamination also having been investigated. Trends in the level of pollutants are simpler to detect in sediments due to the fact that they are not subject to the same fluctuations as pond surface water which is driven by precipitation or influxes of water.^43^ Where possible, to more accurately assess the occurrence and fate of MNPs or TWPs in ponds, a combination of water and sediment matrices enables more robust analysis of the efficacy of ponds; however, only 18% of studies take a multi-matrices approach, with 53% analysing only pond sediment and 29% analysing only water (Table 1).

**Table 1:** Detailed information of pond matrix studied, contaminant studied, analytical methods, and results. Contaminant quantification units are taken directly from studies with the heterogeneity of units reflecting the lack of standardisation in the study of MNPs and TWPs in pond systems

Author	Pond type	Pond location	Catchment	Matrix	Target contaminant category	Polymer type	Contaminant quantification	Organics removal and density separation	Analytical instrumentation	Particle size range or minimum size as determined by filters	Co-occurring or leached contaminants
Ashiq *et al.*, 2023	Detention pond	Memphis, USA	Residential and commercial	Sediment	MP		Pond 1: 37 517 MP per kg DW; pond 2: 54 082 MP per kg DW	Organics removal: 30% H_2_O_2_ density separation: CaCl_2_ (density 1.38 g cm^−3^)	Fluorescence microscope	10–750 μm	
Brooks *et al.*, 2023	Stormwater pond	Florida, USA	Residential, highway and commercial	Water and sediment	MP	Polyethylene (PE), polypropylene (PP), polystyrene (PS), polyethylene terephthalate (PET), or other	Pond 1: 0.0–55.5 items per m^3^ in water 2.5–203.0 items per kg dry weight in sediment	Density separation: NaCl (density 1.2 g mL^−1^) and NaI (density 1.7 g cm^−3^)	Visual sorting and Raman microscope	<1 mm, 1–5 mm, >5 mm (300 μm net)	
							Pond 2: 0.0 to 11741.5 mg m^−3^ in water 0 to 5313.0 mg kg^−1^ DW in sediments				
Camponelli *et al.*, 2009	Stormwater retention pond	Baltimore County, USA	Highway	Sediment	TWP						Zn (as a proxy for TWP)
Corcoran *et al.*, 2025	Wet retention ponds and one dry pond	London, Canada	Residential and industrial	Sediment	MP	Polyethylene (PE), polypropylene (PP), acrylics, alkyds, polyurethane (PU), polyvinyl chloride (PVC) and polyethylene terephthalate (PET), epoxy, polyacrylonitrile, acrylonitrile and nylon	Fifteen ponds studied.	Density separation: sodium polytungstate (density 1.5 g cm^−3^)	Microscope and FTIR	53 μm to 2 mm	
							Lowest average: 0.7 ± 0.7 g^−1^ dry weight in sediment				
							Highest average: 2322.5 ± 4642.8 g^−1^ dry weight in sediment				
							All pond average: 163.52 ± 324.5 g^−1^ dry weight in sediment				
De Oliveira *et al.*, 2024	Infiltration pond	Nantes, France	Highway	Sediment	TWP		At the inlet of the pond SBR and BR = 2.06 ± 0.07 mg g^−1^		Py-GCMS	Small diameter (63–160 μm), larger diameters (200–500 μm)	As, Cd, Cr, Cu, Li, Mo, Ni, Sb, V, Zn
							At the middle of the pond SBR and BR 4.99 ± 0.21 mg g^−1^				
							At the overflow section SBR and BR = 7.05 ± 0.29 mg g^−1^				
Kadir *et al.*, 2025	Stormwater ponds	Sydney, Australia	Urban and commercial	Water	MP	Polystyrene (PS), polyethylene (PE), chlorinated polyethylene (CPE), high-density polyethylene (HDPE), polypropylene (PP), poly(ethyl methacrylate) (PEMA), poly(vinyl stearate) (PVS), polyurethane (PUR), polyvinyl chloride (PVC), polyester (PES), polyethylene terephthalate (PET), and nylon	Model yacht pond: 3.3 to 10.5 particles per L	Organics removal: 30% H_2_O_2_	uFTIR	>20 μm and <5 mm	
							Musgrave pond: 4.5 to 10.1 particles per L	Density separation: NaCl (density 1.2 g cm^−3^)			
							Willow pond: 3.1 to 11.9 particles per ^−1^				
Kumayon *et al.*, 2023	Retention ponds	Saskatoon, Canada	Residential	Water	MP	PET, polyethylene terephthalate; PP, polypropylene; PS, polystyrene; Unc., uncharacterized	6.44 ± 3.6 particles per m^−3^	Organics removal: Fenton oxidation 0.25 g FeSO_4_ and 12.5 mL H_2_O_2_	Raman microscope	10 μm net	
								Density separation: NaI (density 1.6 g cm^−3^)			
Liu *et al.*, 2019a	Retention ponds	Denmark	Residential, industrial, commercial and highway	Sediment	MP	PE, PS, PVC, PP, PA, PU, polyester, ABS, epoxy, acrylic, SAN, vinyl copolymer, EVA, PVAC, polycarbonate, phenoxy resin, EPDM, acrylic paint, PU paint, and alkyd	8894 items per kg	Organics removal: 30% H_2_O_2_, 50% H_2_O_2_ then Fenton oxidation FeSO_4_	FPA-based mFTIR (<500 μm) and ATR FTIR (>500 μm)	10 μm steel mesh	
								Density separation: ZnCl (density 1.97 g cm^−3^)			
Liu *et al.*, 2019b	Retention ponds	Denmark	Residential, industrial, commercial and highway	Water	MP	PE, PS, PVC, PP, PA, PU, polyester, epoxy, PVAC, acrylic, SAN, vinyl copolymer, EVA, PVA, phenoxy resin, alkyd and acrylic paints	490–22 894 items per m^3^	Organics removal: 50% H_2_O_2_ and Fenton oxidation using 146 mL 50% H_2_O_2_,63 mL of 0.1 M FeSO_4_ and 65 mL of 0.1 M NaOH	FPA-based mFTIR (<500 μm) and ATR FTIR (>500 μm)	10–2000 μm	
							85–1143 μg m^−3^	Density separation: ZnCl (density 1.7 g cm^−3^)			
McKenzie *et al.*, 2024	Retention ponds	North East Scotland	Road	Water	TWP				UHPLC-MS		Bisphenol-A, 1*H*-benzotriazole, 5-methylbenzotriazole, tetrabromobisphenol-A, 1,2,5,6,9,10-hexabromocyclododecane, *N*,*N*-diphenylurea, 1-cyclohexyl-3-phenylurea, 3-cyclohexyl-1,1-dimethylurea, *N*,*N*-dicyclohexylmethylamine, 2-(methylthio)benzothiazole, 2-aminobenzothiazole, 2-hydroxybenzothiazole, 1,3-diphenylguanidine, benzothiazole, 2-(4-morpholinyl)benzothiazole, 2-mercaptobenzothiazole, 2,4,6-tris[bis(methoxymethyl)amino]-1,3,5-triazine, *N*-cyclohexyl-1,3-benzothiazol-2-amine, dimethylphthalate, diethylphthalate, benzylbutylphthalate, di-*n*-pentylphthalate, *N*-(1,3-dimethylbutyl)-*N*′-phenyl-*p*-phenylenediamine quinone, 9,10-dihydro-9,9-dimethylacridine, *N*-cyclohexyl-*N*′-phenyl-*p*-phenylenediamine
Moruzzi *et al.*, 2020	Detention reservoir	Sao Paulo, Brazil	Urban	Sediment	MP and TWP	PE, PES, PP, PS, PA (polyamide), black particles identified as TWPs	57 542 units per kg	Density separation: ZnCl^2^ (density 1.6 g cm^−3^)	Microscope and ATR FTIR, Raman microscope	(<0.5 mm, between 0.5 mm and 1 mm and >1 mm); (<0.5 mm) represented 89% of the total MPs, and this category mainly included fragments (62%) and pellets (30%)	
Öborn *et al.*, 2024	Retention ponds	Sweden	Residential, commercial and highway	Sediment	MP and TWP	PP (12), polyester (11), PE (10), polyurethane (PU) (9), PS (7), acrylic (7), PVC (6), PA (3), epoxy/phenoxy resins (3), SBR (3) acrylonitrile butadiene styrene (ABS) (1) and cellulose esters (1)	MP count: 1440–77 200 items per kg	Organics removal: Fenton oxidation 50% H_2_O_2_, 0.1 M FeSO_4_, 5 M NaOH	uFTIR	20 μm steel filter	
							Average: 17 600 items per kg				
							Median: 7070 items per kg				
							MP mass 120–2950 μg kg^−1^	Density separation: ZnCl^2^ (density 1.82 g cm^−3^)			
							Average: 1180 μg kg^−1^				
							Median: 858 μg kg^−1^				
Olesen *et al.*, 2019	Wet retention pond	Viborg, Denmark	Urban	Water, sediments, and vertebrate fauna	MP	Acrylonitrile butadiene styrene (ABS), acrylic, acrylic paints, alkyd, aramid, cellulose acetate, diene elastomer, ethylene propylene diene monomers (EPDMs), epoxy, ethylene vinyl acetate (EVA), polyamide (PA), polyacrylonitrile (PAN acrylic), polyethylene (PE), Pebax®, polyethylene glycol (PEG), phenoxy resin, polylactic acid (PLA), polycarbonate, polyester, polyoxymethylene (POM), polypropylene (PP), polystyrene (PS), polytetrafluoroethylene (PTFE), polyurethane (PU), polyurethane paints (PU paints), polyvinyl alcohol (PVA), polyvinyl acetate (PVAC), polyvinyl chloride (PVC), styrene acrylonitrile (SAN), styrene butadiene rubber (SBR), and vinyl copolymer	Water: 270 items per L & 4.2 μg L^−1^	Organics removal: 50% H_2_O_2_ and Fenton oxidation 50% H_2_O_2_, 0.1 M NaOH, 0.1 M FeSO_4_	uFTIR	10–500 μm	
							Sediment: 106 items per kg & 0.4 g kg^−1^	Density separation: ZnCl (density 1.7 g cm^−3^)			
Parker-Jurd *et al.*, 2024	Retention ponds	UK	Highway	Water and sediment	TWP		Water (TWP): influent 4.1 mg L^−1^ ± 3.22, effluent 0.22 mg L^−1^ ± 0.13, sediment (TWP): 3.83 mg g^−1^ ± 0.89		Py-GCMS	Min 15 μm	
							Water (MP): road runoff 4.63 mg L^−1^ ± 4.83, effluent 2.32 mg L^−1^ ± 1.93, sediment (MP): 4.22 mg g^−1^ ± 3.84				
Rasmussen *et al.*, 2024	Stormwater retention ponds	Denmark	Industrial (including plastic recycling) and residential	Sediment and water	MP and TWP	Other, PVC, polyester, PU, PS, PP, PE	Water 0.3 × 10^0^–1.2 × 10^3^ counts per L	Organics removal: 30% H_2_O_2_ and Fenton oxidation 50% H_2_O_2_, 0.1 M FeSO_4_	Py-GCMS and FPA-FTIR		
							0.2 × 10^0^–4.2 × 10^2^ μg L^−1^				
							Sediments 6.5 × 10^1^–3.5 × 10^4^ counts per g and mass 5.3 × 10^0^–1.8 × 10^4^ μg g^−1^	Density separation: sodium polytungstate (density 1.95 g cm^−3^)			
Upadhyay & Bajpai, 2024	Retention pond and natural park pond	Chhattisgarh, Raipur, India	Industrial and residential	Water	MP	PE, PS, PA, PP, PET, other, SAA, ABS, IR-PC, PMMA, PVC, EVOH, EPDM	2.52 ± 1.28 particles per L for bucket samples and 2.93 ± 1.34 particles per L for net samples	Organics removal: Fenton 30% H_2_O_2_ 0.05 M FeSO_4_	Microscope FTIR ATR;	50 μm–5 mm	
Wik *et al.*, 2008	Detention pond	Western Sweden	Road	Sediment	TWP	PP and PE, PS, PVC, PA and PET TWP	0.15 to 10.8 g kg^−1^			10 μm to 4550 μm	

Sample collection, preparation, analysis and quantification methods vary by matrix type and study. However, even within these matrix classifications there is no standard operating procedure for sampling, making comparisons between studies problematic. Where the depth of water sample collection was described, they ranged from 20 cm below the surface,^44,45^ 0–30 cm below the surface,^46^ 50 cm below the surface^47^ to “just below the surface”.^48^ The sampling depth is directly relevant to the retention mechanisms of balancing ponds with the roles of sedimentation and contaminant density primary considerations in particulate transfer. The volume of water samples also varied markedly between studies from 30 L (ref. 46) to 46 220 L.^43^ Similarly, the depth of sediment sampled varied from the top 1–2 cm (ref. 13) to as deep as 20 cm.^49^

#### 3.2.2 Sample preparation

Generally, for both water and sediment the removal of organic matter and density separation were undertaken but with methods differing between studies. To remove organic matter H_2_O_2_ and/or Fenton oxidation was often used but with varying compositions or concentrations (Table 1). Where density separation was carried out a variety of solutions were used including NaCl (mass density 1.2 g cm^−3^), NaI (mass density 1.7 g cm^−3^, 1.6 g cm^−3^), ZnCl_2_ (mass density 1.97 g cm^−3^, 1.7 g cm^−3^, 1.6 g cm^−3^, 1.82 g cm^−3^), CaCl_2_ (mass density 1.38 g cm^−3^), and sodium polytungstate (SPT) (mass density 1.5 g cm^−3^, 1.95 g cm^−3^) (Table 1). The variety of solution mass densities used for density separation has implications for which polymers and elastomers will separate and as such which are captured for analysis. Lower density solutions are insufficient to separate high density polymers such as polyvinyl chloride (PVC) or polystyrene (PS) from the matrix, thus potentially losing critical data from results. Both PVC and PS are amongst the six most widely fabricated polymers^23^ and the six most widely used commercial polymers.^50^

#### 3.2.3 Sample analysis

Once samples were prepared, the quantification of contaminants was undertaken using several methods: primarily microscopy, spectroscopy and thermoanalytical techniques (Table 2). Although early analysis of TWPs looked towards using Zn as a proxy for TWPs,^51,52^ studies post 2019 use a variety of methods including Fourier-transform infrared (FTIR) and Raman spectroscopy, microscopy and/or pyrolysis gas chromatography mass spectrometry (Py-GCMS) for TWP or MNP analysis depending on the target contaminant (Table 2). Stereomicroscope analysis is also occasionally undertaken but principally as an additional analytical step to count MPs and select a proportion of these for further analysis using polymer characterisation techniques such as FTIR.^53–55^ Py-GCMS (thermoanalytical) techniques derive mass results and FTIR/Raman (spectroscopy) primarily delivers MP particle count data with some studies using software to extrapolate these data to estimate mass information, *e.g.* Rasmussen *et al.*^45^ This difference in quantification immediately makes comparisons between studies difficult. Both thermoanalytical methods and spectroscopy can determine polymer type, but microscopy techniques also enable the determination of polymer size and shape. The size and number of particles are important for determination of ecosystem and human health effects, with smaller nanoparticles being able to passively cross the cells of humans and aquatic species.^56^ Shape and size have also been highlighted as a determinant of the transfer of contaminant through the pond system.^43^ FTIR allows MNP polymer type to be identified by acting as a source of infrared radiation sending specified IR wavelengths through the sample causing some of the sample molecules to vibrate, and increasing vibrational energy at the expense of radiation energy causing absorption of IR radiation at certain frequencies. The absorption provides a molecular “fingerprint” which enables the identification of polymers by matching the fingerprint with libraries.^57^ Raman is also a type of vibrational spectroscopy used to investigate the chemical and physical characteristics of polymers. Raman determines polymer type based on the scattering of light by the sample polymer causing the wavelength of the scattered photons to change, creating a fingerprint that can be matched with polymer libraries.^58^ However, due to the absorption of light through the infrared spectrum by black TWPs the use of FTIR and Raman techniques are not effective in the characterisation or quantification of TWPs.^59^ Neither FTIR nor Raman is currently sensitive enough to identify smaller plastics.^58,60^ This limitation has restricted the minimum polymer size of samples analysed in ponds to 10 μm, although Raman has identified polymers as small as 1 μm in non-environmental samples.^61^ Raman spectroscopy does have several further limitations including a relatively weak signal necessitating prolonged sample exposure times, the potential for laser damage to samples as a result of direct photodegradation or sample heating, and a proneness to spectral distortion induced by fluorescence.^62–64^ Fluorescence can result in a raised baseline which can completely overshadow the Raman signal driven by factors such as the composition of the MP, impurities such as colouring agents, and degradation products.^63^ Due to this the surface of the polymer must be clean for analysis,^58^ making organic removal critical in the sample preparation. As already discussed, there is no consistency in studies relating to organic removal, highlighting the possibility of differences in results due to sample preparation and therefore further compounding the difficulty in study comparisons. Fluorescence interference can also be mitigated by the use of metal-coated polycarbonate filters to optimize the contrast between the filter and the MPs with metals including gold, nickel, titanium, and aluminum used to coat the filter paper;^65^ however, this significantly increases analysis costs. Multiple filter types again make comparisons between studies more difficult. Nevertheless, Raman spectroscopy has a wider spectral coverage and is more adept at identifying differences between polymers and is less sensitive to moisture than FTIR,^60,62^ making it more useful for polymer identification and characterization for samples taken from matrices seen in ponds.

**Table 2:** Microplastic, nanoplastic and tyre wear particle analysis capabilities

Analytical instrumentation	Microplastic analysis	Nanoplastic analysis	Tyre wear particle analysis	Polymer shape analysis	Polymer size analysis	Particle count	Mass	Polymer type	Destructive
Py-GCMS	Yes	Yes	Yes	No	No	No	Yes	Yes	Yes
IR	Yes	No	No	Yes	Yes	Yes	*Via* software calculation	Yes	No
Raman	Yes	No	No	Yes	Yes	Yes	*Via* software calculation	Yes	Potential laser damage
Microscope (inc. SEM and TEM)	Yes	Yes	Yes	Yes	Yes	Yes	No	No	No

Thermoanalytical methods used in the study of MNPs and TWPs in ponds are focused upon the use of Py-GCMS. Although sample preparation methods for Py-GCMS may be approached in a similar way to spectroscopic sample preparation,^66^ unlike spectroscopic methods, Py-GCMS can be used to analyse black particles, most notably TWPs as well as MNPs. Py-GCMS provides a mass of contaminant rather than the count data produced by spectroscopy methods, again making comparisons between studies difficult. The pyrolysis unit of the Py-GCMS rapidly heats the sample whereby the resulting gaseous products are injected into a separatory column connected to the MS. When analysing a variety of polymers and elastomers most researchers employ moderate pyrolysis temperatures to avoid a decreased polymer signal; in the study of TWPs and MNPs in ponds pyrolysis temperatures range from 600 °C to 610 °C.^13,45,67^ The pyrolysis behaviour of most MNPs and TWPs is predictable; when pyrolyzed the products or “marker compounds” are identifiable by their mass spectra.^66^ These marker compounds are used to calibrate the Py-GCMS to enable mass quantification. However, the availability of appropriate analytical or calibration standards is a further challenge with standard operating procedures for the analysis of TWPs largely lacking. Alongside mass quantification one of the primary benefits of Py-GCMS is that there is no lower size limit to detection, thus enabling the analysis of nanoplastics alongside microplastics and TWPs. The limitation to the determination of size becomes sample preparation rather than analytical capability.

### 3.3 Micro(nano)plastic and tyre wear particle characteristics in ponds

#### 3.3.1 Particle type

Alongside TWPs, the most fabricated polymers are polypropylene (PP), low-density polyethylene (LD-PE), high-density polyethylene (HD-PE), polyvinyl chloride (PVC), polyethylene terephthalate (PET), and polystyrene (PS).^50^ With the exception of PET, these are also the most reported polymers found in pond studies. PP is the most prevalent contaminant in terms of both count and mass where TWPs were not determined. However, TWPs dominate the share of particle load in sediments when MPs and TWPs are considered together.^45,54,67^ The density of polymers is often not reported in studies, notwithstanding its importance as a critical characteristic in terms of the density separation step in sample processing. Despite this, particle density is often highlighted as a rationale for MP abundance in sediment and relative scarcity in the water column. Even so, polymers that have a greater density than water are still found to be present in the water column. Wik *et al.*^51^ found PVC, with a density greater than that of fresh water, to be the most prevalent polymer in the water column. Studies also demonstrate the presence of polymers that have a density less than that of fresh water, such as PS, PP, and PE, in sediments (Table 1), indicating that density is not the only driver for retention in ponds. Brooks *et al.*^43^ demonstrated that 76.5% of polymers in sediments had a density lower than that of water and highlighted the role of biofilms and aggregation in MP transport and deposition. This study also found that PE was the most prevalent polymer in both sediment and water. Similarly, Liu *et al.*^59^ and Öborn *et al.*^68^ found a high volume of low-density polymers in pond sediment, with PP being the most commonly found polymer, again indicating that polymer density was not the only factor in sedimentation.

#### 3.3.2 Particle size

Rasmussen *et al.*^45^ highlight that in addition to polymer density individual polymer size could aid preferential sediment capture with ponds retaining larger particles with a higher settling velocity rather than smaller particles. Conversely, smaller polymer size has been determined to decrease retention through sedimentation, increase leaching of toxins and increase transport through ponds.^13,59^ However, despite the link between size fraction, sedimentation, leaching and transport, the smallest fraction size discussed in the reviewed studies is 10 μm, an order of magnitude more than the start of the nanoplastic classification (Table 1).

Polymer size is discussed in a number of studies in relation to the retention of MPs by sedimentation, particularly through the application of Stokes' law. Stokes' law is a mathematical formula from fluid dynamics that expresses the settling velocity of a single spherical particle through Newtonian fluid. According to Stokes' law this settling velocity is dependent on particle diameter, particle density, fluid density and viscosity. However, particles must be perfectly spherical, smooth, and rigid in shape.^69^ This is an obvious limitation in the prediction of naturally aged MNPs and TWPs which are often not spherical in shape. Liu *et al.*^44^ highlighted that due to Stokes' law large particles have a tendency to either float to the water surface or to settle to the bottom of the pond and noted the importance of large particle size in conjunction with polymer density to the increased concentration of particle count in sediments. Polymers with a larger particle size were also measured in higher concentrations than smaller particles by Olesen *et al.*^47^ and statistical difference (*p* < 0.05) was demonstrated between particle size in sediments and particle size in water columns, with larger particle size evident in sediments. However, Olesen *et al.* also note that some of the larger particles found in the sediment should have floated according to Stokes' law, again indicating that MP capture by sediments cannot be explained simply by sedimentation. Ingestion and subsequent egestion by zooplankton or vertebrates creating pellets with higher density was postulated as a reason for a change in density and subsequent sedimentation. Hydraulic mixing was also suggested as an explanation of the presence of smaller buoyant MPs in sediments with convective currents causing them to come into contact with these sediments and become immobilized. The formation of biofilm on the surface of the particle can also promote sedimentation even for lower density polymers.^59^

It is also acknowledged that smaller MPs can more easily be transported by the water cycle.^54^ Transport in and potentially through the ponds is a key element to understanding the retention of MNPs and TWPs by ponds. De Oliveira *et al.*^13^ described size fraction as being critical to understanding the fate and spatial distribution of TWPs, with smaller particles being more prevalent at the pond outflow. This was attributed to faster settling of coarser particles as compared to finer particles and redistribution of finer particles driven by turbulence created by rainfall intensity. The tendency for ponds to preferentially retain larger denser particles rather than smaller less dense particles was also noted by Rasmussen *et al.*^45^ The study highlights that whilst Stokes' law does not precisely predict the settling of all particle sizes and shapes, it does set out a general trend that “floating” plastics, determined by size and mass, are more likely to stay in the water column and as such have the potential to leave the outflow of a pond. However, despite the acknowledgement of the importance of smaller sized particles in the movement through and therefore potentially out of ponds, NPs are not specifically mentioned in any study. The inability of prevalent analytical techniques such as Raman spectroscopy to detect environmental plastics below ∼10 μm is likely to have hindered critical analysis of plastics and TWPs in the nano fraction and thus omitting the size fraction that is potentially the most damaging to ecosystems and human health.

#### 3.3.3 Particle shape

Alongside polymer density and size, polymer shape has also been discussed in relation to movement and sedimentation. The importance of polymer shape highlights a key weakness of Py-GCMS analysis inasmuch as this destructive analytical method cannot record shape, unlike spectroscopy methods. Due to the prominence of Py-GCMS in terms of TWP analysis it also removes a key discussion point in studies that review the occurrence, fate and transport of these particles. Despite the limitations on TWP shape analysis, Parker-Jurd *et al.*^67^ highlight that TWPs are typically elongated in shape. Outside of TWPs, the particle shapes that are most often discussed include fragments, films, fibres and pellets with fragments generally dominating particle counts in both sediments and water (Fig. 2).^46,49,55,59^ Although fragments, films and fibres are prevalent in sediments, Brooks *et al.*^43^ suggest that circular, round, smooth particles tend to settle before irregular-shaped particles. Conversely, Ashiq *et al.*^49^ suggested that the smooth shape of pellets found in sediments is more likely attributed to processes that smooth fragments over time as a consequence of retention within the sediment rather than their circular, round, smooth shape contributing to retention. Whilst shape is an important parameter it is discussed in less detail than size or density, with questions such as the effect of MP shape on toxicity being unanswered.^54^ A fuller understanding of the mechanisms that drive the retention of MNPs and TWPs by ponds is required to maximise retention efficacy through the design of man-made ponds.

**Fig. 2:**
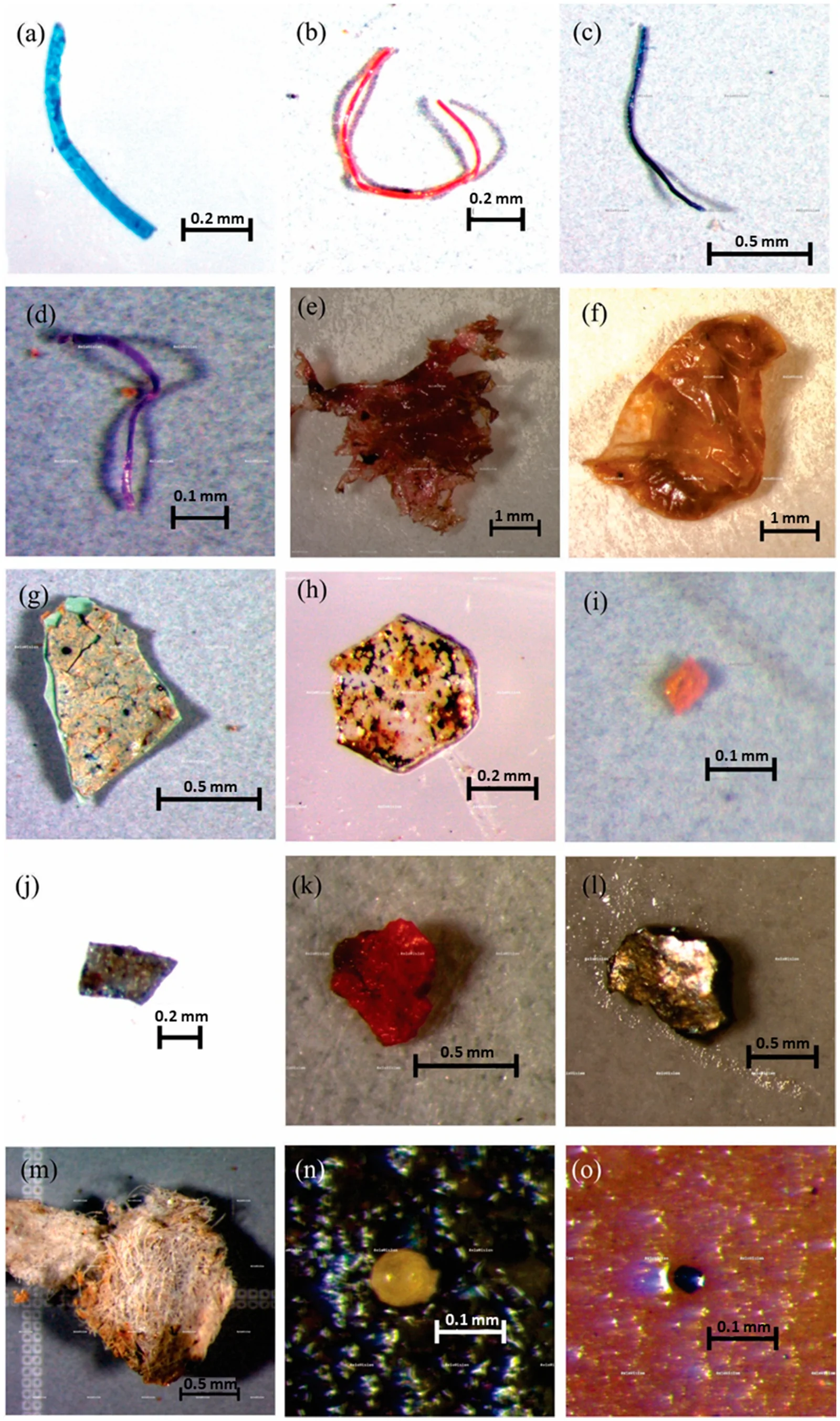
Shapes of MPs found in sediments from a stormwater drainage reservoir, Sao Paulo, Brazil: (a–d) lines/ﬁbres; (e–h) ﬁlms and sheets, (i–m) fragments; and (n and o) pellets.^54^

### 3.4 Efficacy of ponds in the retention of micro(nano)plastics and tyre wear particles

Whilst the determination of the presence and quantity of MNPs and TWPs is an important part of this research, the true value of the study of ponds is gaining an understanding of their role in the retention of these contaminants. However, accurately quantifying inputs and outputs from ponds is a difficult process requiring a consistent sampling regime over a sustained period of time. As a result, many studies do not study the efficacy of ponds in the retention of MNPs and TWPs but rather either discuss the movement of particles within, but not out of, the pond as a proxy for retention or quantify the presence of these contaminants in the relevant matrices. In the few studies where efficacy through mass balance of particles is considered, approaches are based on estimations and assumptions rather than a rigorous input and output sampling regime over time and even here, they do not explicitly consider the role of nanoparticles.

#### 3.4.1 Pond efficacy – particle inflow and outflow estimations

The studies that most accurately determine the retention of MPs and TWPs in ponds are those that attempt to estimate particle inflow and outflow concentrations. Although endeavours at such estimations are sparse, De Oliveira *et al.*^13^ do discuss efficacy through mass balance calculations of a pond in Nantes, France. These calculations rely on estimating TWP emissions per vehicle per km, traffic density, time frame, and the catchment of the pond in question. Whilst the catchment data can be ascertained from planning documents, and the traffic density was calculated from traffic data collected locally to the pond in 2019,^70^ the estimation of TWPs provides a larger level of uncertainty. Estimations of tyre wear emissions must take into account variables such as driving style, vehicle type, and climate.^71^ The increase in electric vehicles (EVs) with greater weight and torque also leads to increased tyre wear emission, and as a result the proportion of EVs must also be taken into account.^72,73^ A combination of these factors means that uncertainties with the inflow estimations presented by De Oliveira *et al.* must be recognised. This inflow estimation was then compared against the mass of TWPs retained in the pond sediment to give a mass balance. TWP proxy concentrations were determined to be 2.06 ± 0.07 mg g^−1^, 4.99 ± 0.21 mg g^−1^ and 7.05 ± 0.29 mg g^−1^ for the entrance, middle and overflow sections, respectively, signifying a 3.4-fold increase in TWP concentration from entrance to overflow. Alongside sedimentation, particle size was highlighted as a key factor in the movement of TWPs with finer sediment found towards the outflow as a result of faster settling of larger particles at the inflow and turbulence at the inflow remobilising finer particles. This again raises the question around the fate of nanoparticles and the efficacy of ponds in their retention. Although the volume of TWPs increased from the inlet to the outlet, the pond was seen as an effective sink for TWPs. Due to the nature of the pond, water column measurements were not taken and the outflow TWP concentrations were not measured.

Olesen *et al.*^47^ also calculated the efficacy of a pond in Viborg, Denmark, using mass balance calculations. These estimations were built on precipitation data, catchment size, inflow approximations, assumptions that the concentration of MPs in the water column would be mirrored in concentrations of MPs exiting the outflow, and assumptions that the concentrations of MPs in the sediment were constant over time. These assumptions resulted in an estimation that 85% of MPs were retained by the pond with 401.5 mg kg^−1^ MPs in sediments, 4.2 μg L^−1^ MPs in the water column, and 12.9 mg kg^−1^ MPs in fauna. Olesen *et al.* recognised that the uncertainty of the assumptions behind these calculations is unknown and probably quite large but that the data nevertheless show that ponds are important sinks for MPs.

Rasmussen *et al.*^45^ also undertook mass balance calculations using different methods, estimations and assumptions to those of both DeOliveira *et al.* and Olesen *et al.* Although rainfall intensity and runoff volumes were measured using flow meters and rain gauges to determine runoff volume received by the pond, runoff inflow was not sampled and analysed for MNPs or TWPs. Rather, sediment cores were sampled throughout the pond, and pond water was sampled at the outlet of the pond. The accumulated inflow of MPs and TWPs in the sediment plus the TWPs and MPs suspended in pond water collected close to the outlet were determined to be the mass of particles that had entered the system. The removal efficiency was calculated as the accumulation of MPs and TWPs minus the MPs and TWPs suspended in water near the outflow. Using this mass balance calculation a removal efficiency between 83.9% and 95.3% for small MPs (less than 500 μm), between 80.1% and 100.0% for TWPs, and between 90.7% and 99.9% for large MPs (over 500 μm) was reported. A lack of direct measurement of MPs and TWPs both into and out of the ponds is acknowledged by Rasmussen *et al.* and was attributed to study time and resource limitations. A lack of direct measurement of MPs and TWPs into and out of the pond necessitates reliance on a number of assumptions with associated uncertainties. The presence of these uncertainties must be acknowledged when considering the removal efficiency data presented by Rasmussen *et al.*

Parker-Jurd *et al.*^67^ calculated removal percentage by determining the TWP concentration in pond first flush influent followed by the determination of MP concentration in effluent during the same rainfall event in multiple ponds in the Midlands and Southwest of the UK. The subsequent subtraction of effluent from influent resulted in an average removal efficiency of 77% of TWPs with influent water concentrations containing an average of 4.1 mg L^−1^ TWPs ± 3.22 (0.17–29.8 mg L^−1^), and effluent concentrations in the range of 0.011–1.21 mg L^−1^ (average 0.22 ± 0.13). However, Parker-Jurd *et al.* highlight that neither the TWP flux into or out of the pond was determined directly due to such methods being time- and resource-intensive as inflow and its concentration vary substantially over time.

Each of the studies that incorporate particle inflow and outflow concentrations of MPs and/or TWPs does so through different methods, further complicating comparisons between studies. This is exacerbated by the fact that efficiency estimations from each study do not measure the inflow or outflow concentrations of MPs or TWPs but rather employ calculations with assumptions leading to recognised limitations. The time- and resource-intensive nature of an accurate inflow and outflow determination of MPs and TWPs is often cited as the rationale for this omission.

#### 3.4.2 Pond efficacy – particle progression or presence

The progression of MPs and TWPs through the pond system, determined by concentration at varying points in the pond, is a further method used to estimate pond efficacy. In these studies, change in MP or TWP concentration or mass is recorded at or near any pond inlet and at or near any outlet. From here assumptions are made about the efficacy of ponds in the removal of MPs or TWPs without inflow or outflow concentrations of MPs and/or TWPs. Öborn *et al.*^68^ used this method by taking sediment cores at ponds in Sweden to determine an average MP concentration of 1260 μg kg^−1^ at inlets and 1090 μg kg^−1^ at outlets and an average TWP concentration of 20 700 μg kg^−1^ at inlets and 3300 μg kg^−1^ at outlets. The reduction in concentration and particle count was used as a proxy to determine the efficacy of ponds as MNP and TWP sinks.

Ashiq *et al.*^49^ used a similar method to determine the efficacy of ponds as a sink in Memphis, USA, by collecting sediment samples at two ponds at multiple inlets and outlets. Samples were taken near the confluence of inlet drains and at the farthest outlet from the inlets to determine longitudinal spatial distribution of MPs. The average mass concentration of MPs in surface soil was seen to decrease from 265.13 mg kg^−1^ to 19.91 mg kg^−1^ inlet to outlet in pond 1 and 236.41 mg kg^−1^ to 215.49 mg kg^−1^ in pond 2. However, the particle count increased from inlet to outlet by 11% in pond 1 and 22% in pond 2. The increase in MPs at the outlet was driven by smaller MPs moving towards the outlet, again raising the question of the distribution and fate of NPs in the pond systems and the efficacy of the retention of these particles by ponds. Ashiq *et al.* attribute the greater retention of larger particles closer to the inlets in the hyporheic zone to the interaction of surface flow with the bed following traditional colloid filtration theory. This theory states that particle size-dependent retention processes such as straining, where particles are trapped in pore throats which are narrower than the size of the particles, retain larger particles but allow smaller particles to move through the pond system.

The presence and therefore retention of MPs and TWPs in sediment is also used as an indicator that ponds can trap some of the MPs and prevent them from being transported downstream.^59^ This method concentrates on the volume or mass of trapped MPs or TWPs as evidence of the efficacy of ponds as sinks rather than any mass balance estimations or particle progression from inlet to outlet.

### 3.5 MNPs and TWPs as a source of chemical additive leaching, a vector for contaminants, and potential health and environmental consequences

The presence of MNPs and TWPs potentially have extensive environmental and health consequences which are exacerbated by their sorption and concentration of other contaminants as well as their leaching of potentially toxic manufacturing additives. However, the efficacy of ponds as a sink for MNPs and TWPs alongside other contaminants or leachates is not widely studied. Although the impact of MNPs and TWPs on human health is currently not fully understood correlations between the presence of these particles and deleterious effects on the nervous system, kidney system, respiratory system, digestive system, and placenta are evident.^5^ Combined exposure to other contaminants such as heavy metals, PFAS, and PAHs alongside MPs has been seen to augment the negative effects of these contaminants, with increased accumulation and particle retention in terrestrial and aquatic organisms enhancing toxicity, transference along the food chain and attendant human health issues.^28,74,75^ Considering MNPs and TWPs as a vector for sorbed contaminants and additives in the context of ponds is a crucial topic which is not sufficiently explored in the current literature. Whilst the type, size and shape of these particles alongside their presence in ponds are often discussed, the characterisation of toxic additives leached by these particles and (in)organics (de)sorbed by MNPs and TWPs, such as PFAS, PAHs, or metals, is not. The efficacy of ponds as a sink for MNPs and TWPs cannot be fully ascertained without an understanding of what happens to leachate products and sorbed contaminants.

De Oliveira *et al.*^13^ at the time of writing is the only study to acknowledge and measure the presence of contaminants co-occurring with TWPs in ponds. The study considers pond efficacy within the framework of a multi-contaminant system by considering TWP interaction with metals and potential environmental sorbents such as organic matter in pond sediment. Metals such as Zn, Cu, Cd, As, and Cr were seen to correlate to TWPs with this relationship attributed to either the pre-existence of metals in tyre composition or the sorption of metals by TWPs. The sorption of metals by MNPs is not studied in the existing suite of literature examining ponds as a sink for MNPs despite MNPs being recognised as vehicles for sorbing and transporting (post) transition metals from urban surfaces to inland waters.^76^ The abrasion suffered by LDPE and PET as a result of mechanical interactions in a silt/sand synthetic stormwater has been seen to increase Pb and Zn sorption and subsequent release in laboratory studies.^77^ Sorption by MPs in a silt/sand synthetic stormwater matrix has also been seen to reduce the uptake of (post) transition metals by sediment.^76^

Whilst it is recognised that MNPs can act as vectors for co-occurring pollutants and pathogens with potentially significant ecological and health consequences,^78^ the consideration of environmental parameters, such as natural organic matter (NOM) or pH in the (de)sorption of co-occurring pollutants in pond systems is currently lacking. Laboratory work and studies in other aquatic environments indicate that NOM routinely found in pond sediments^79^ is seen to influence the (de)sorption of co-occurring contaminants. Environmental factors such as humic acid, fulvic acid, and extracellular polymeric substances can stabilise MNP suspensions, thus reducing metal ion release through a combination of oxygenated functional groups promoting complexation and the aromaticity of NOM facilitating aggregation.^80^ The role of pH in sorption is also well understood but not discussed in current MNP/TWP pond literature. Mechanisms related to the sorption of inorganics onto negatively charged sorbents such as MNPs are driven in part by pH with increases in pH driving an increase in the negative charge of functional groups on sorbents promoting inorganic sorbent complexation and ion exchange.^81,82^ Conversely higher pH reduces the sorption of negatively charged contaminants to negatively charged sorbents, such as MNPs and TWPs, through electrostatic repulsion with increasing pH driving desorption.^5,83^ The role of a potentially changing pH in pond systems and how this relates to the (de)sorption of a variety of contaminants is also critical to understanding (de)sorption in the pond system and needs to be investigated in future studies.

The kinetics of (de)sorption is a further facet that should be explored within the context of pond field studies going forward. Knowledge of the kinetic features of MNP interactions with co-occurring contaminants is fundamental for the determination of water quality, yet studies of MNPs and TWPs in ponds do not consider this study area.^84^ Sorption kinetics of MNPs and TWPs in primarily laboratory contexts have been seen to be strongly influenced by particle size, aging, and temperature. Log *K*_d_ values in relation to organic contaminants are generally understood to increase with decreasing particle size from MP to NP;^85^ however, sorption has been reported to decrease with decreasing particle size within the NP size range.^86^ Similarly with inorganic sorption smaller MNP particle size has been associated with greater sorption.^87^ Desorption experiments indicate that the stronger interactions between smaller MPs and contaminants impede pollutant desorption, thereby increasing the persistence and mobility of pollutant-loaded MPs in aquatic environments.^88^ If ponds are true sinks for MNPs and TWPs the retention of smaller particles could also indicate the retention of sorbed contaminants; however, pond studies indicate that smaller particles are more likely to be transported through and out of the pond systems where there is more potential for them to enter the food chain, bioaccumulate and desorb in gastric fluid.^89^ Although pond studies examine various size ranges of MPs and TWPs the smallest particle size considered is 10 μm. To fully incorporate the potential effect of MNPs and TWPs in and beyond pond systems a greater focus on (de)sorption in conjunction with particle transport though ponds is necessary. Laboratory studies have also highlighted the importance of aging on MNP and TWP (de)sorption. Increased surface area and oxidation induced by artificial UV aging has been seen to increase sorption and desorption of co-occurring contaminants over shorter periods, suggesting greater contaminant release into the environment.^89,90^ The length of time that MNPs and TWPS are retained in ponds is directly relevant to the desorption and release of sorbed contaminants. Laboratory studies have also shown that increases in temperature lead to an increase in desorption of contaminants from MNPs, indicating that warm environments could promote greater contaminant release with attendant consequences on surrounding ecosystems.^90^ The interplay between MNPs, TWPs, and (de)sorbed contaminants should be considered in conjunction with environmental parameters within the context of ponds to determine pond efficiency.

McKenzie *et al.*^91^ is the only pond study to highlight the number of additive chemicals that can leach into water from TWPs such as DPG, benzothiazoles, 6PPD and its transformation product 6PPD-Q. The presence of these chemicals was measured as above regulatory limits and were not seen to decrease as they moved through the pond system, indicating the ponds ineffectiveness at the capture of these TWPs additives. River studies have demonstrated the toxicity of exposure to the antioxidant 6PPD-Q in particular, which was seen to lead to acute mortality in coho salmon.^32^ The leaching of TWP additives has also been evidenced in the study of sedimentation tanks that are designed to collect road runoff from tunnels. Tunnels are enclosed environments that act as a sink for TWPs and MPs before runoff transports the particulates to sedimentation tanks where they are retained in a manner akin to ponds. Rødland *et al.*^92^ and Meland *et al.*^93^ both reported an increase in the concentration of leached additives in tank outlets with an increase in particle retention time due to the leachability and mobility of these additives. Additives such as 6PPD-Q, DPG, 2-hydroxybenzothiazole (OHBT), benzothiazole-2-sulfonic (BTSA), and 2-aminobenzothiazole (ABT) were seen to rise above regulatory thresholds whilst particles were retained, demonstrating that the retention of TWPs and MNPs does not necessarily equate to successful remediation. It should be noted that whilst these sedimentation tanks are equitable to ponds in terms of high retention times for MNP and TWP particles, they do not expose these particles to UV in the same way as pond systems. UV is well documented as a key driver of MNP and TWP aging leading to leaching of manufacturing additives.^94^ Nevertheless, the leaching of additives in sedimentation tanks does allow some parallels to be drawn with ponds and strongly indicate the potential for additive leaching from particles in the pond system, particularly when particles are held in sediments deprived of UV. In similar ways to (de)sorption, particle size and aging are primary factors in the leaching of additives by TWPs and MNPs.^94,95^ In their study using TWP leaching in collected runoff Weyrauch et al^96^ observed leaching kinetics for key toxic additives such as DPG and 6PPD-Q. Kinetics were seen to follow two phases, a rapid first phase leaching followed by a slower second phase. The half-lives for DPG were seen to be 17 days in phase one and 250 days in phase two, and for 6PPD-Q were seen to be 80 days for phase one and 520 days for phase two. Of the chemicals studied 6PPD-Q was seen to be the most stable with 15–30% remaining in the TWPs after two years of the biodegradation experiment. Weyrauch *et al.* surmise that the volume and concentration of environmental TWPs from roads with such a long half-life indicate the potential for the build-up and release of additives at environmentally significant concentrations. In contrast, Foscari *et al.*^97^ also undertook TWP leaching laboratory studies utilising road runoff and sediment basin treated road runoff as their mediums. This study suggests far quicker leaching as a result of environmental parameters including microbial degradation and surmises that this may suggest that ponds may be effective in the reduction of chemicals released by TWPs. These contrasting findings from laboratory studies examining road runoff, which constitutes the primary input to pond systems, highlight the need for further leaching studies to be conducted within the specific context of pond systems. Leaching and (de)sorption kinetics and transport should be considered in conjunction with MNP and TWP pond kinetics. The settling velocities of sediment particles outside of MNPs and TWPs is well studied with factors such as particle size distribution, temperature (with increased temperatures increasing settling velocities), and inflow velocities forming key constituents of model calculations to determine sediment residence time. However, studies reviewing pond MNPs and TWPs have not modelled their progression through ponds. Once such modelling has taken place specifically focusing on MNPs and TWPs, optimisation of pond design can then be considered taking into account pond design factors such as pond shape, length-to-width ratio, depth and location of inlets and outlets^98^ to mitigate both particulate and leachate (de)sorption contamination.

No published pond study assesses the leaching of additives from MNPs or measures the concentration of contaminants sorbed by MNPs and subsequent leaching. Although the existing body of literature that considers the presence of plastic additives in larger freshwater systems is relatively underdeveloped it does exist. The presence of endocrine-disrupting additives such as diethylhexylphthalate (DEHP), organophosphate esters (OPEs), phthalates (PAEs) and bisphenols (BPs) linked to plastic food containers and textiles has been evidenced in rivers, indicating the importance of leaching studies in ponds.^99–101^

## 4. Future direction

The importance of ponds as a potential sink to hamper the transfer of TWPs and MPs from the terrestrial to the aquatic is apparent. However, existing studies rarely consider the role of TWPs and MNPs as a vector, there is no standardisation of analysis, TWPs are often not considered, and the role of NPs is entirely missing. These areas should be addressed in future studies.

TWPs should be routinely included in future pond contaminant studies. The importance of the analysis of TWPs present in the influent, effluent, water column and sediment of ponds is apparent with between 50% and 70% of the polymers and elastomers released environmentally being determined as TWPs.^10,12^ The pond studies that do separate TWPs and MPs determine TWPs to represent ∼47% of polymers and elastomers,^67^ a proportion that necessitates inclusion.

The exclusion of NPs from research into plastics and ponds is also apparent. There is evidence of increased progression through pond systems for smaller MPs. Studies need to address whether this inverse relationship between progression and size continues into the nanoscale. Such nanoscale particles have been determined to be less likely to precipitate out of the water column than MPs, enhancing the likelihood of transport through ponds and into waterways, and to leach additives at many orders of magnitude greater than the leaching rate of MPs.^102^ An understanding of whether NPs are transported out of ponds into waterways has the potential to enable the study and implementation of preventative measures. The move towards the consideration of NPs can be facilitated by extending the use of Raman to sub-10 μm particles, as has been achieved in non-environmental samples, to pond samples.

The additives associated with TWPs and MNPs are acknowledged as toxic with marked environmental consequences.^32^ Despite this observation, the effects of TWP and MNP leachate from particles either retained in or transferring through ponds are very rarely studied. The potential for MNPs and TWPs to be retained in ponds but leach additives which may be transferred through ponds into waterways is a pathway that future research needs to consider. The efficacy of ponds cannot be fully assessed without taking a holistic view of what is retained by the pond be it TWPs, NPs, additive leachate or contaminants sorbed by TWPs or MNPs. McKenzie *et al.*^91^ demonstrate the analytical capability to measure additive leaching through UHPLC is already available to undertake this assessment. These techniques should be used in future studies.

For these research areas to form the future direction in the study of the efficacy of ponds in retaining TWPs and MNPs there needs to be an underpinning provided by standardised analytical techniques. Comparison between research studies is problematic due to differing analytical approaches used for quantification, varying minimum size of MPs and TWPs, and studies reporting count or mass. Standardisation of sampling techniques and analytical protocols would result in comparisons between research being more effective. The adoption of two consistent unit reporting needs to be adopted, one for particle count per kg (or L in aqueous systems) and one for concentration at mg kg^−1^ (or L in aqueous systems).

## 5. Conclusion

Ponds have traditionally been used as a method of improving water quality with their effectiveness in mitigating the transport of MPs and TWPs into waterways considered in recent years. Despite the potential of ponds to be used as a sink for MNPs and TWPs the number of published studies in this research field are limited. Where research has been undertaken the efficacy of ponds has been estimated to be between 83.9% and 99.9% for MPs and 80.1% and 100.0% for TWPs, where caveats around the potentially substantial uncertainty of these estimates should be acknowledged. However, there are a number of significant gaps in the current body of research such as the transport and fate of NPs, the intermittent inclusion of TWPs, and the consideration of the leaching of manufacturing additives and co-occurring contaminants. As analytical techniques develop, future studies should ensure that these aspects are considered more thoroughly. Standardisation of this reporting would also enable robust study comparison and the drawing of relevant conclusions that could shape the design of future pond systems.

## Author contributions

Stuart Cairns: conceptualization, formal analysis, methodology, investigation, writing – original draft. Muhammad Junaid: writing – review and editing. Iain Robertson: writing – review and editing.

## Conflicts of interest

The authors have no relevant financial or non-financial interests to disclose.

## Data Availability

As a review article there are no original data pertaining to this manuscript. Rather any data are to be found in the papers that have been cited.
